# P-568. Transmitted Drug Resistance to Antiretroviral Therapy in Chile: Preliminary Results from a Cross-Sectional Study

**DOI:** 10.1093/ofid/ofae631.766

**Published:** 2025-01-29

**Authors:** Maria Elena Ceballos, Cinthya Ruiz-Tagle, Felipe Castañeda, Marcela Ferrés, Carlos Palma, Angelica Domínguez de Landa, Manuel Espinoza, Alejandro Afani, María Elvira Balcells

**Affiliations:** Pontificia Universidad Católica de Chile, Santiago, Region Metropolitana, Chile; Pontificia Universidad Católica de Chile, Santiago, Region Metropolitana, Chile; Pontificia Universidad Católica de Chile, Santiago, Region Metropolitana, Chile; Pontificia Universidad Católica de Chile, Santiago, Region Metropolitana, Chile; Pontificia Universidad Católica de Chile, Santiago, Region Metropolitana, Chile; Pontificia Universidad Católica de Chile, Santiago, Region Metropolitana, Chile; Pontificia Universidad Católica de Chile, Santiago, Region Metropolitana, Chile; Hospital Clínico Universidad de Chile, Santiago, Region Metropolitana, Chile; Pontificia Universidad Católica de Chile, Santiago, Region Metropolitana, Chile

## Abstract

**Background:**

The use of antiretroviral therapy (ART) has reduced HIV morbi-mortality and its transmission. However, treatment failure can occur when acquiring a strain with mutations conferring resistance to antiretrovirals. Transmitted resistance to antiretroviral drugs (TDR) reported nationally was 10.45% in 2018 and is increasing worldwide. We aimed to determine the percentage of global TDR and the relevance of incorporating the genotyping study in naïve people living with HIV in Chile.

**Figure:**
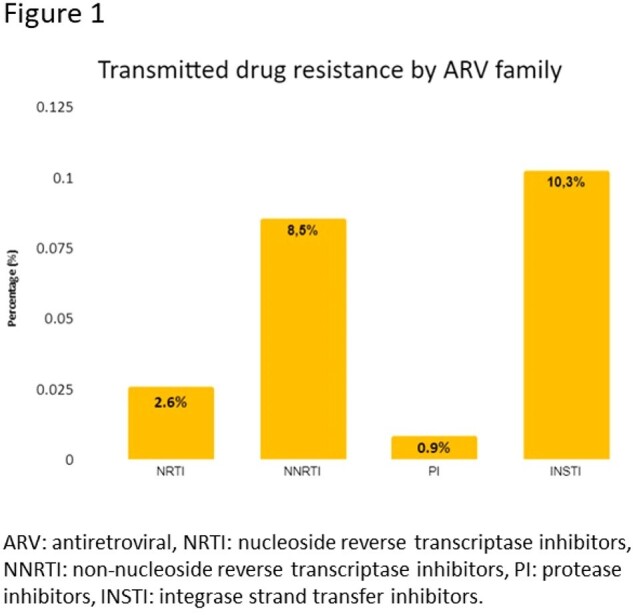

**Methods:**

Observational, cross-sectional study conducted in 11 healthcare centers located in 7 regions of Chile. Individuals ≥18 years old with an HIV diagnosis in the last 12 months and without prior ART were invited to participate. Individuals with an HIV viral load < 1000 ARN copies/mL were excluded. ARN genotyping of the reverse transcriptase, protease and integrase genes were carried out using a nested PCR with subsequent Sanger sequencing. The percentage of individuals with TDR was determined and mutations associated with TDR were identified according to the HIV Drug Resistance Database, Stanford University.

**Figure:**
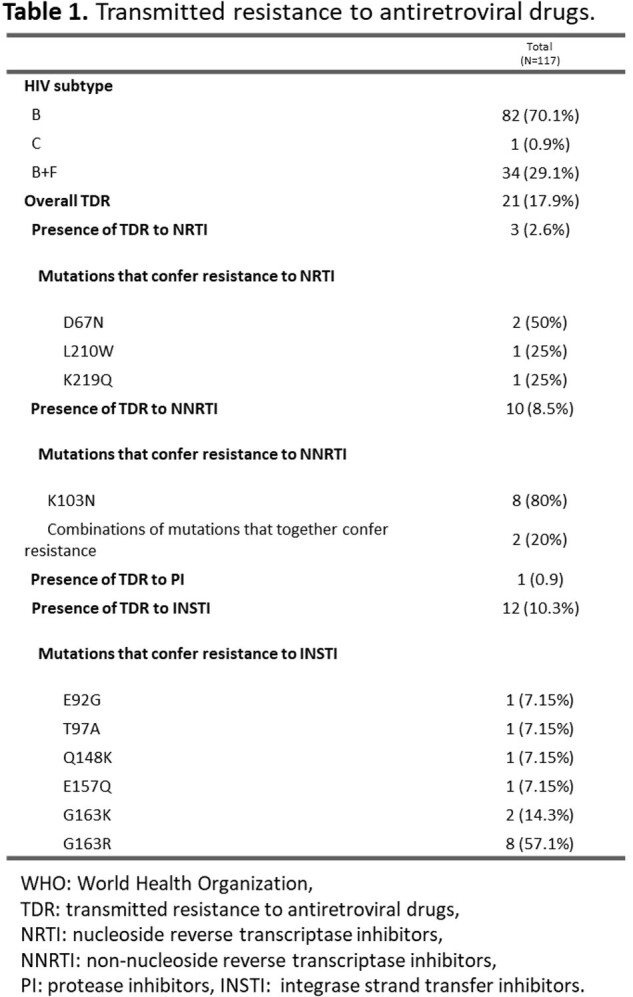

**Results:**

Between February 2023 and April 2024, 117 participants have been sequenced (final sample size 164). The average age is 35.2 years (18-78), with 90.6% belonging to the Chilean population. 87.2% are male, of which 74.4% have sex with men. Most common viral strain was subtype B (70.1%). Overall TDR (TDR to any ART family) was 17.9% (n=21). TDR for each family and mutations are shown in Table 1 and Figure 1. In addition, other mutations were found, but they were not included in the percentage for TDR because they are not in WHO TDR list (Table 2). Three participants presented resistance to more than one ART family; one to NRTI and NNRTI, one to NRTI and INSTI and one to NNRTI and INSTI.

**Figure:**
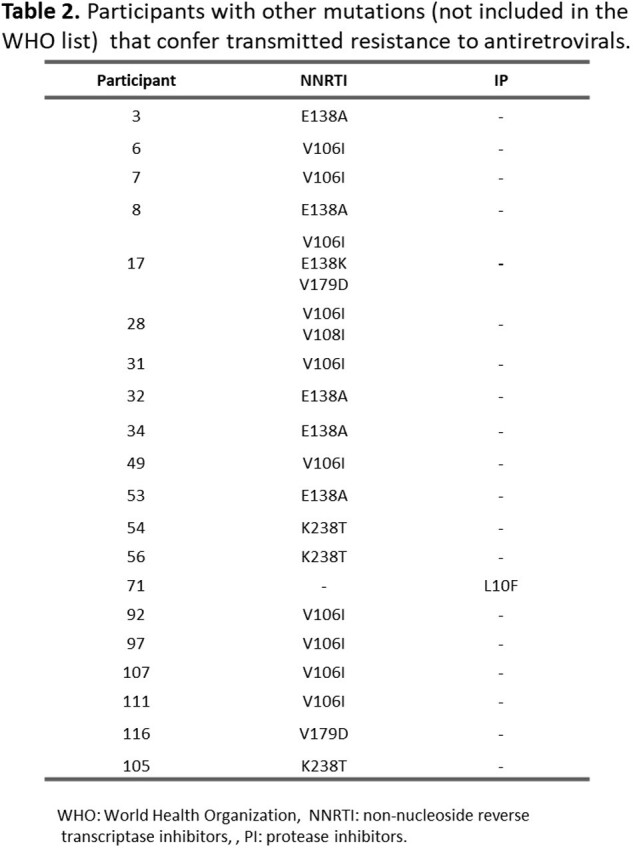

**Conclusion:**

In Chile, an increase is observed in the percentage of TDR to ART compared to what has been historically reported. The main families affected are the NNRTIs and the INSTIs (mostly first generation). Considering these preliminary results, it is considered pertinent to incorporate the baseline genotyping study in patients starting ART with both NNRTI efavirenz or rilpivirine and first-generation INSTI.

**Disclosures:**

**All Authors**: No reported disclosures

